# Therapeutic Options for Chronic Rhinosinusitis in N-ERD Patients

**DOI:** 10.3389/falgy.2021.734000

**Published:** 2021-08-24

**Authors:** Rik J. L. van der Lans, Wytske J. Fokkens, Sietze Reitsma

**Affiliations:** Department of Otorhinolaryngology, Amsterdam University Medical Centres, University of Amsterdam, Amsterdam, Netherlands

**Keywords:** NSAID (non-steroidal anti-inflammatory drug), N-ERD, chronic rhinosinusitis, aspirin desensitization, aspirin therapy, biological

## Abstract

Patients with non-steroidal anti-inflammatory drug (NSAID)-exacerbated respiratory disease (N-ERD) often suffer from chronic rhinosinusitis (CRS) with nasal polyps, a form of primary diffuse Type 2 CRS. Although this disease is also seen in NSAID-tolerant patients, CRS in N-ERD often is more severe and more treatment resistant; local nasal therapy (nasal corticosteroids) and endoscopic sinus surgery are employed like in NSAID-tolerant patients, but with limited and/or short-lived effects. This mini-review gives an overview of the current additional treatment options for CRS in N-ERD. As such diets, aspirin therapy after desensitization, antileukotriene therapy and biologicals are discussed based on the current body of literature. Selecting the right treatment strategy depends on shared-decision making, local availability and cooperation between ENT-surgeons, allergists, and pulmonologists.

## Introduction

Chronic rhinosinusitis (CRS) is an inflammation of the nose and paranasal sinuses lasting for more than 12 weeks, and leading to nasal obstruction, rhinorrhea, loss of smell, and/or facial pain/pressure (1). This condition is common in most of the world, with a questionnaire-based prevalence ranging from 5 to 28% (2–4). Adding nasal endoscopy and/or imaging to confirm the diagnosis, reduces the prevalence to 3–6% (5–7). The societal burden of CRS is immense, and mostly dictated by indirect costs (absenteeism and presenteeism) (8, 9).

CRS symptoms are quite bothersome, leading to a significant reduction in health-related quality of life (10), especially when nasal polyps are present (CRSwNP), and even more in those suffering from non-steroidal anti-inflammatory drugs (NSAID)-exacerbated respiratory disease (N-ERD) (11, 12). The estimated prevalence of N-ERD related disease within the CRS population varies from 9.6 to 16%. The reported prevalence depends, amongst others on the population studied (general population vs. tertiary referral centers), differences between countries, and methodologies used (questionnaire-based vs. provocation-based) (13–15).

Patients with CRS and N-ERD are at risk for uncontrolled sinonasal disease, requiring more surgeries and/or more intense medical therapy, often with unsatisfactory results (15). This review aims to give an update of the current therapeutic options for the management of CRS in patients with N-ERD.

## Pathophysiology and Classification of CRS in N-ERD Patients

The pathophysiology of N-ERD is incompletely understood. It appears that in hypersensitive patients, a baseline mast cell activation state is present. Upon exposure to NSAIDs, this is further triggered by decreased prostaglandin E2 synthesis through inhibition of cyclooxygenase-1, and subsequent activation and chemotaxis of inflammatory cells (such as basophils and eosinophils) through mast-cell derived prostaglandin D2 (16). As a result, nasal polyps show vast amounts of eosinophils and evidence of Type 2 inflammation, such as elevated levels of IL-5 and IL-13 in nasal secretion (17).

In the past decades, CRS was divided between “with polyps” (CRSwNP) or “without polyps” (CRSsNP) based on the endoscopic appearance of polyps. However, with the newest update of the European Position Paper on Rhinosinusitis and Nasal Polyps 2020 (EPOS2020), a more diverse classification is proposed (1). CRS is first classified as primary or secondary, based on the absence or presence of underlying pathology such as cystic fibrosis. Primary CRS is then divided between localized or diffuse disease, and endotyped roughly as either Type 2 or Non-type 2 disease. The CRS found in N-ERD patients thus classifies as primary diffuse Type 2 CRS. We will use this latter terminology in the rest of this review, although most literature is based on the former (simplified) diagnosis of CRS(wNP).

## Appropriate Medical Therapy and Fess; Limitations in N-ERD

Following the management schemes of EPOS2020 the first line of treatment for primary diffuse CRS is appropriate medical therapy (AMT), consisting of, but not limited to local corticosteroids (either spray, drops, or rinses), saline rinses and/or oral corticosteroids. Those achieving disease controls with AMT should be advised to continue their medication without the need for further investigation or therapy. However, when patients indicate a poor disease control with AMT, it is advised to perform additional investigations in order to differentiate the disease as a Type 2 or Non-type 2 primary diffuse CRS. As described above, patients with CRSwNP and N-ERD fulfill the criteria for Type 2 disease.

Generally, the additional treatment options for primary diffuse Type 2 CRS are the addition of oral corticosteroids to AMT (if not tried before) or functional endoscopic sinus surgery (FESS). The long-term aim of performing FESS is to open up the paranasal sinuses in such a way that they are more accessible for local therapy, ideally leading to better disease control with AMT. Short-term goals include the removal of diseased mucosa and polyps, directly alleviating symptoms such as nasal obstruction and fullness. FESS in itself is an umbrella term and does not necessarily describe the extent of surgery. A FESS for diffuse Type 2 CRS can range from a simple polypectomy (removing polyps from the nasal cavity) to the opening of all the paranasal sinuses (maxillary, ethmoidal, sphenoidal and frontal approach) which is often termed “full house FESS.” Surgery can further be extended, for example by additional approaches to the maxillary sinus (such as a medial maxillectomy, removing the inferior nasal turbinate, and associated lateral nasal wall) or extended drilling procedures to the frontal sinuses (Draf III or “modified Lothrop” procedures). These extensive options challenge the “functional” approach in FESS and oftentimes authors refer to endoscopic sinus surgery, or ESS, to cover all possible (endonasal) approaches. Furthermore, ESS can be extended by aggressive removal of all sinus mucosa (so-called “reboot surgery”). As neatly summarized in EPOS2020, a debate is ongoing on the needed extent of surgery and as yet, no firm conclusions can be drawn on the added value of more aggressive approaches (18–20).

Studies show that especially patients with N-ERD show a limited effect of AMT and/or FESS, resulting in higher oral corticosteroid use, more frequent surgery and a lower quality of life (15, 21). Qualitative analyses show that patients experience great frustration as they often find themselves trapped between their allergist suggesting more oral corticosteroids and their ENT-surgeon advocating yet another surgery (22). As such, many N-ERD patients have to deal with a chronic, yet uncontrolled disease state of their CRS. It is therefore pivotal that treating physicians acquaint themselves with the additional treatment options listed below.

## Drug Avoidance and Diet

N-ERD patients are advised to avoid NSAIDs, selective COX-1 inhibitors, and alcohol consumption (21) as these might trigger a sudden increase in symptoms from the upper and lower airways, asthma exacerbation, bronchospasm or even death. COX-2 inhibitors are generally well-tolerated. We are not aware of studies reporting on the success of such avoidance stratagems or how often patients are (unintentionally) exposed to these drugs or triggers.

Another possible option would be to consider a low-salicylate diet. A cross-over randomized-controlled trial performed in 30 patients (14 of which originating from a smaller trial), showed an improvement of all evaluated items including sinonasal disease-specific quality of life (22-item SinoNasal Outcome Test; SNOT-22), nasal complaints, nasal endoscopy scores, and asthma control (23). These results were confirmed in a questionnaire-based study with 30 N-ERD patients following a low-salicylate diet for 2 weeks. SNOT-22 scores decreased significantly and the difference was relevant (mean difference 12 points) (24). It should be noted, however, that there was no placebo group, numbers were small and a large portion of participants had mild CRS based on baseline SNOT-22 scores.

A different diet with high omega-3/low omega-6 fatty acids showed promising result in a small pilot trial with 10 adult patients suffering from primary diffuse Type 2 CRS and N-ERD (25). Along with biomarkers indicating a change in cellular fatty acid composition, SNOT-22 scores decreased 15.1 points on average (95% CI: −24.3; −6.0) after a 2-weeks high omega-3/low omega-6 fatty acids diet. This unblinded setup might contain placebo effects and the findings remain to be confirmed in larger studies.

## Aspirin Therapy After Desensitization

Aspirin therapy after desensitization (ATAD) is based on a two-step treatment: first, patients are desensitized by exposure to increasing doses of oral aspirin; second, they continue using high doses of aspirin daily. Several studies have investigated this treatment option in primary diffuse Type 2 CRS in N-ERD patients.

The finding that N-ERD patients experience a short period (24–72 h) of symptom alleviation after a challenge with aspirin, and that they are refractory to additional aspirin challenges (26), has led to the development of several desensitization protocols. A widely used protocol aims at oral aspirin 625 mg twice daily after a startup phase (27). There have been several studies describing the effect of such protocols on CRS signs and symptoms, four of which are placebo-controlled trials.

A small study with 27 CRS patients undergoing ATAD (28), showed significant improvement of nasal symptoms, such as nasal congestion, discharge, and overall discomfort. The visible amount of polyps did not improve significantly. Moreover, only 12 of the original 27 patients were available for evaluation as 5 had ATAD treatment complications and 10 chose to discontinue ATAD. Similar findings were obtained in a small prospective trial with 12 patients of whom 8 were still on maintenance therapy after 6 months, with comparable endoscopic scores to baseline, but improved symptom scores as measured with the SNOT-22, decreasing from a mean of 30.0 at baseline to 18.5 after 6 months (29). A study with 30 post-operative CRS patients showed a sustained improvement of endoscopic scores compared to baseline, due to surgery, and further improving SNOT-22 scores during aspirin treatment after surgery up to 30 months of follow-up (30). Another retrospective study of post-operative ATAD showed similar results in 34 patients, of whom 2 could not complete the desensitization phase and 5 stopped in the maintenance phase due to gastrointestinal or respiratory side-effects (31). Apart from these (and other) small studies, four double-blind placebo-controlled trials have been published on the use of ATAD for CRS in N-ERD patients (32–35), three of which were used for a meta-analysis in EPOS2020 (1). ATAD was shown to have a significant effect over placebo in the reduction of SNOT-22 scores or other symptoms scores. However, this did not reach the threshold for a clinically relevant difference. The lung functions scores (FEV-1) were significantly better with ATAD. The meta-analyses were based on relatively small pooled patient groups, ranging between 70 and 85 patients.

The downsides of ATAD include side-effects. Some patients are not able to endure the desensitization phase, others experience respiratory or gastrointestinal problems. Other medical conditions that require elective surgery can also be a cause of discontinuation. Furthermore, the medication regime is strict and not a single day of aspirin can be missed in order for ATAD to be successful. Current evidence, in line with the studies described above, shows that for a relatively large portion of N-ERD patients, ATAD is not a long-term solution (36).

To conclude, ATAD is a relevant treatment option for primary diffuse Type 2 CRS in N-ERD patients. Patient selection, education and shared decision making are key for a successful application of this treatment.

## Antileukotrienes

Leukotrienes are a class of breakdown products of arachidonic acid produced by eosinophils and mast cells. There might be a role for these leukotrienes in the pathophysiology of primary diffuse Type 2 CRS in general; especially in the context of N-ERD, there seems to be a more direct link. Nasal polyp biopsies from N-ERD patients show an increased number of leukocytes expressing leukotriene receptors as compared to non-N-ERD nasal polyps, with a reduction in these leukocytes after a desensitization protocol (37). Therefore, it seems logical to use antileukotriene drugs, such as montelukast or zileuton, in the treatment strategy of CRS in general, and specifically in N-ERD patients.

After reviewing the current evidence on antileukotrienes in CRS, the EPOS2020 steering committee concluded that the use of such drugs in CRS was not advised, as (limited) evidence shows no benefit over the use of nasal corticosteroid or when used in combination with nasal corticosteroids. Only in patients not tolerating nasal corticosteroids, one might consider antileukotrienes although there a no supporting studies (1).

Apart from the evidence reviewed by EPOS2020, two studies have been performed to address the effect of antileukotrienes for primary diffuse Type 2 CRS in the specific context of N-ERD. One study evaluating montelukast contained 33 N-ERD patients who were allocated to use either intranasal corticosteroids, montelukast, or both, directly after FESS. After a follow-up of 12 months, no differences were found in nasal endoscopy scores of the polyps, or patient-reported outcome measures (visual analog scale (VAS) and SNOT-22) (38). The other study retrospectively analyzed post-operative patients using zileuton (*n* = 18) vs. those without (*n* = 27), showing no differences in clinical outcomes over an average follow-up of 2.8 and 2.4 years, respectively (39). Of note, the zileuton group used this drug for 77 days on average (range 6–300 days). Although these two studies are small and from their setup give limited evidence, it seems that also for N-ERD patients specifically, antileukotrienes are not to be recommended as an additional treatment strategy for their CRS.

It is important to recognize, however, that the European Academy for Allergy and Clininal Immunology (EAACI) position paper on the diagnosis and management of N-ERD does describe the use of antileukotrienes in N-ERD patients as add-on therapy for their asthma (21). As such, a N-ERD patient with both CRS and asthma might benefit from these drugs, but the indication should be derived from the lower airway disease status.

## Biologicals

With the advent of biological therapy, the management of primary diffuse Type 2 CRS has been tremendously revolutionized. Pivotal trials and the first real-life data show rapid disease control and improvement of the quality of life, often beyond the effect sizes that treatment strategies hitherto could offer (40–42). However, these trials target patients with primary diffuse Type 2 CRS *per se*, either in the context of N-ERD or in NSAID-tolerant patients. No randomized-controlled trials with N-ERD patients only have been performed.

### Omalizumab

Omalizumab is a monoclonal antibody directed against immunoglobulin E. It has been approved as add-on therapy for CRSwNP in 2020. Previously, it has already been used for over a decade by pulmonologists for the treatment of asthma. Therefore, many (small) reports on the effects of omalizumab for N-ERD focus on the lower airways, with a SNOT-22 as marker for the upper airways at best (43). One open study with 16 N-ERD patients receiving omalizumab vs. 16 N-ERD patients with ATAD, describes improvement of CRS signs and symptoms in 14 of the 16 omalizumab patients. Together with a decrease in patient-reported scores, there was a reduction of nasal polyp scores on a scale from 0 to 8: baseline mean score 3; after 9 months the mean score was 0, indicating no visible polyps left. In the ATAD group, nasal polyp scores remained unchanged (mean score 3 at baseline and 2.9 at 9 months) (44).

### Dupilumab

Dupilumab is a monoclonal antibody directed against the alpha unit of the IL4/IL13 receptor. It was approved late 2019 as add-on therapy for CRSwNP. In a *post-hoc* analysis from a previously published phase 2a trial (45), the N-ERD patients (n=19) and NSAID-tolerant patients (*n* = 41) were compared. Both groups showed significant improvements in disease control after 16 weeks of treatment, as assessed by imaging (Lund-Mackay score), patient-reported outcome measures (SNOT-22 total score, SNOT-22 sense of smell/taste score), and smell test score (University of Pennsylvania Smell Identification Test). Eight N-ERD patients were in the dupilumab-treated group. Their nasal polyp scores were significantly reduced by a mean of 2.51, whereas the NSAID-tolerant patients had a non-significant reduction of 0.72.

### Mepolizumab

Mepolizumab is an antibody directed against IL-5. It is currently not registered for use in CRSwNP patients, but for asthma only. In a retrospective study including 17 N-ERD patients receiving mepolizumab for severe asthma, SNOT-22 scores were observed to decrease significantly and relevantly by 17.7 points (*n* = 11). Specific SNOT-22 questions on nasal congestion and smell/taste also showed an improvement with three or more doses of mepolizumab (46).

### General Comments

Although there are hardly any specific studies on the effect of biologicals for CRS in the context of N-ERD, it is highly likely that these patients will follow the observations seen in NSAID-tolerant Type 2 CRS patients. In a recent meta-analysis on biologicals for CRS, all three biologicals with at least phase 2a data were shown to be effective. Direct comparisons were hard to make due to differences in study setup, patient population, treatment duration, etc. No clear advice could be given on which biological to prefer (47).

In practice, the use of biologicals in CRS, and therefore in N-ERD patients, is a matter of shared-decision making, depending on the local situation/availability, physician experience, and patient preference.

## Future Needs

In order to better understand and compare treatment effects, and to facilitate meta-analyses, we would advise authors of future studies on primary diffuse Type 2 CRS to report effects in N-ERD patients separately. It remains to be elucidated whether the presence of N-ERD represents a clinically relevant subgroup when it comes to treatment outcomes, or that most patients with primary diffuse Type 2 CRS in the context of N-ERD respond largely similar to NSAID-tolerant patients.

## Discussion

This mini-review describes the possible therapeutic options for primary diffuse Type 2 CRS in N-ERD patients. Apart from the customary appropriate medical therapy and surgery, ATAD and especially biologicals hold most promise for the future.

The treatment options are summarized in Table 1. For most of the therapeutic options described, patient counseling and shared-decision making are key to success. Whether one discusses diets, ATAD, or biologicals, all of these represent a long-term therapy, needing long-term treatment adherence. Treating physicians should be keen to inquire for patient preferences, past experiences and future hopes. As such, clear communication with the patient is essential, with an important role for managing expectations. Furthermore, a patient-tailored approach includes treatment plans for the upper airway (as discussed here), and for the lower airways as well. Most of these treatments are therefore best employed in close collaboration between allergists, pulmonologists and ENT-surgeons.

**Table 1:** Summary of treatment options for primary diffuse Type 2 CRS in N-ERD.

**Treatment**	**N-ERD specific?**	**Evidence**	**Remarks**
AMT	No	+++	Cornerstone treatment for any diffuse CRS
(F)ESS	No	+++	Debate on the role of extent of surgery
Drug avoidance/diets	Yes	+	Small studies
ATAD	Yes	+++	Benefits lower airways as well; beware of side-effects and high discontinuation rates
Antileukotrienes	No	–	Indication might be derived from lower airways; no (good) evidence for treatment of CRS
Biologicals	No	+++	Dependent on local situation/availability

*CRS, chronic rhinosinusitis; N-ERD, non-steroidal anti-inflammatory drugs exacerbated respiratory disease; AMT, appropriate medical therapy; (F)ESS, (functional) endoscopic sinus surgery; ATAD, aspirin therapy after desensitization*.

Unfortunately, the evidence for most treatment options relies on relatively small studies. Therefore, studies targeting N-ERD patients specifically are needed, and studies including both NSAID-tolerant and intolerant patients should report on the subgroup of N-ERD subjects separately. Especially biologicals deserve attention in the coming years, not only with a focus on treatment outcomes, but also on costs. It might well be that in general, biologicals will prove not to be cost-effective in a broad group of primary diffuse Type 2 CRS patients, but only in the more severe cases; N-ERD patients typically fall in the latter group.

## Author Contributions

SR performed the literature search and wrote the initial manuscript. RL and WF reviewed and updated the manuscript. All authors contributed to the article and approved the submitted version.

## Conflict of Interest

RL, WF, and SR received research grants by Sanofi and Novartis. WF and SR have been employed as consultant for Sanofi, Novartis, and GSK.

## Publisher's Note

All claims expressed in this article are solely those of the authors and do not necessarily represent those of their affiliated organizations, or those of the publisher, the editors and the reviewers. Any product that may be evaluated in this article, or claim that may be made by its manufacturer, is not guaranteed or endorsed by the publisher.
